# The Effect of Amoxicillin in Adult Patients Presenting to Primary Care with Acute Cough Predicted to Have Pneumonia or a Combined Viral-Bacterial Infection

**DOI:** 10.3390/antibiotics10070817

**Published:** 2021-07-06

**Authors:** Robin Bruyndonckx, Beth Stuart, Paul Little, Niel Hens, Margareta Ieven, Christopher C. Butler, Theo J. M. Verheij, Herman Goossens, Samuel Coenen, The GRACE Project Group

**Affiliations:** 1 Interuniversity Institute for Biostatistics and Statistical Bioinformatics (I-BIOSTAT), Data Science Institute (DSI), Hasselt University, 3500 Hasselt, Belgium; niel.hens@uhasselt.be; 2 Laboratory of Medical Microbiology, Vaccine & Infectious Diseases Institute (VAXINFECTIO), University of Antwerp, 2610 Antwerp, Belgium; Greet.Ieven@uza.be (M.I.); Herman.Goossens@uza.be (H.G.); samuel.coenen@uantwerpen.be (S.C.); 3 Aldermoor Health Centre, University of Southampton, Southampton SO16 5ST, UK; bls1@soton.ac.uk (B.S.); p.little@soton.ac.uk (P.L.); 4 Centre for Health Economic Research and Modelling Infectious Diseases (CHERMID), Vaccine & Infectious Disease Institute, University of Antwerp, 2610 Antwerp, Belgium; 5 Nuffield Department of Primary Care Health Sciences, University of Oxford, Oxford OX2 6GG, UK; christopher.butler@phc.ox.ac.uk; 6 Julius Centre for Health, Sciences and Primary Care, University Medical Centre Utrecht, 3508 GA Utrecht, The Netherlands; T.J.M.Verheij-3@umcutrecht.nl; 7 Centre for General Practice, Department of Family Medicine & Population Health (FAMPOP), University of Antwerp, 2610 Antwerp, Belgium

**Keywords:** adults, amoxicillin, benefit of treatment, clinical prediction rule, lower respiratory tract infection, pneumonia, primary care, viral–bacterial infection

## Abstract

While most cases of acute cough are self-limiting, antibiotics are prescribed to over 50%. This proportion is inappropriately high given that benefit from treatment with amoxicillin could only be demonstrated in adults with pneumonia (based on chest radiograph) or combined viral–bacterial infection (based on modern microbiological methodology). As routine use of chest radiographs and microbiological testing is costly, clinical prediction rules could be used to identify these patient subsets. In this secondary analysis of data from a multicentre randomised controlled trial in adults presenting to primary care with acute cough, we used prediction rules for pneumonia or combined infection and assessed the effect of amoxicillin in patients predicted to have pneumonia or combined infection on symptom duration, symptom severity and illness deterioration. In total, 2056 patients that fulfilled all inclusion criteria were randomised, 1035 to amoxicillin, 1021 to placebo. Neither patients with a predicted pneumonia nor patients with a predicted combined infection were significantly more likely to benefit from amoxicillin. While the studied clinical prediction rules may help primary care clinicians to reduce antibiotic prescribing for low-risk patients, they did not identify adult acute cough patients that would benefit from amoxicillin treatment.

## 1. Introduction

In Europe, acute cough remains one of the main reasons for consulting in primary care [1]. While most acute cough cases are caused by a self-limiting lower respiratory tract infection (LRTI), general practitioners (GPs) prescribe antibiotics to over 50% [2]. This proportion is inappropriately high given that primary analyses of the Genomics to combat Resistance against Antibiotics in Community-acquired LRTI (GRACE) randomised placebo controlled trial (RCT) found no clear evidence of a clinically meaningful benefit from amoxicillin treatment in adults presenting to primary care with acute cough [3]. In addition, a follow-up analysis demonstrated that there was no clear evidence of a clinically meaningful benefit from amoxicillin treatment in subgroups of patients with LRTI who are most likely to be prescribed antibiotics (e.g., patients with comorbidities, fever, discoloured sputum, etc.) [4]. The subgroups of primary care patients with LRTI which have been shown to benefit from treatment with amoxicillin are limited to patients with evidence of pneumonia on a chest radiograph [5] and patients with a confirmed combined infection (i.e., both a viral and a potential bacterial pathogen detected through modern microbiological methodology) [6].

Routine use of chest radiography and microbiological sampling and laboratory testing are time-consuming and costly, and the results are usually not available when the empiric treatment is prescribed. Therefore, clinical prediction rules which predict these outcomes based on signs and symptoms could be useful to guide antibiotic prescribing in clinical practice. The presence of pneumonia could be predicted in adults presenting to primary care with acute cough through a prediction rule combining the absence of a runny nose with the presence of breathlessness, crackles and diminished breath sounds on auscultation, tachycardia and fever (area under the receiver operating characteristic curve (AUC) 0.70 (95% Confidence Interval (CI): [0.65, 0.75])). When including C-reactive protein (CRP), but not procalcitonin (PCT), the prediction rule’s performance improved (AUC 0.77 (95% CI: [0.73, 0.81])) [7]. Predicting the presence of pneumonia could also be based on the GP’s clinical judgement [8]. The added value of adding CRP or PCT to clinical judgement has not been investigated. Following a similar approach, a prediction rule for the presence of a combined infection could be developed. However, while patients with predicted pneumonia or a predicted combined infection appear to be a good subset for treatment with amoxicillin, the question remains whether or not (the signs, symptoms and biomarkers included in) these clinical prediction rules can identify patients that actually benefit from amoxicillin treatment.

**Figure 1:**
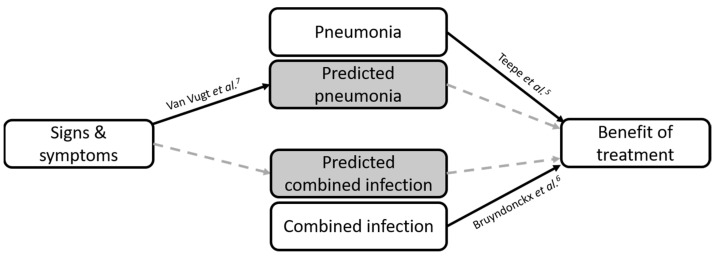
Schematic overview of the objective of this manuscript: full lines are covered in referred manuscripts; dashed lines are covered in this manuscript (5,6,7 in the figure refer to reference [5,6,7]).

Therefore, we set out to assess the effect of treatment with amoxicillin in adults with acute cough who are predicted to have pneumonia or a combined infection (Figure 1).

## 2. Results

**Figure 2:**
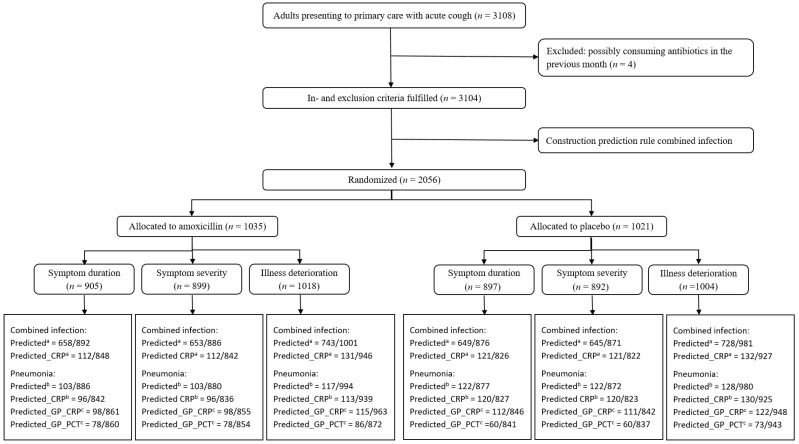
Patient flow chart. ^a^ patients predicted to have a combined infection based on the prediction rule constructed in this manuscript without (predicted) and with (predicted_CRP) inclusion of CRP; ^b^ patients predicted to have a pneumonia based on the prediction rule constructed by Van Vugt et al. without (predicted) and with (predicted_CRP) inclusion of CRP; ^c^ patients predicted to have a pneumonia based on the GP’s suspected diagnosis with inclusion of CRP (predicted_GP_CRP) or PCT (predicted_GP_PCT).

A total of 3108 patients were included in the GRACE study (Figure 2). Four patients that consumed antibiotics in the month before consultation were excluded.

The mean age of patients was 49.8 (standard deviation (SD) 16.8), 40.1% were men and 28.1% were current smokers. Other recorded patient characteristics are presented in Table 1.

### 2.1. Predicting Combined Infection

Microbiology results were available for all patients, identifying a combined infection in 304 patients (9.8%) [9]. Variable importance plots for the imputed datasets are available from the authors on request. The final model contained variables related to general characteristics (duration of prior illness: odds ratio (OR) 0.98, 95% CI: [0.96, 0.99]), patient’s baseline symptoms (runny nose: OR 1.35, 95%CI: [1.02, 1.80] and fever: OR 1.38, 95%CI: [1.08, 1.76]) and clinical signs (prolonged expiration: OR 1.54, 95%CI [1.09, 2.18]). The final clinical prediction rule was based on the pooled parameter estimates and reached an AUC of 0.59 (95% CI: [0.56, 0.63]). Addition of CRP to the final clinical prediction rule increased the AUC to 0.63 (95% CI: [0.59, 0.67]). Addition of PCT to the clinical prediction rule did not change the AUC (0.59 (95% CI: [0.57, 0.63])). Due to the limited added value of including PCT, it was not studied further.

### 2.2. Predicting Pneumonia

Chest radiographs were taken for 2845 patients, of which 2817 were of sufficient quality. Pneumonia was confirmed in 140 (5.0%) of these radiographs. The clinical prediction rule developed by Van Vugt et al. [7] reached an AUC of 0.70 (95% CI: [0.66, 0.75]). Addition of CRP to the clinical prediction rule increased the AUC to 0.79 (95% CI: [0.75, 0.83]). Addition of PCT to the clinical prediction rule increased the AUC to 0.71 (95% CI: [0.67, 0.77]). Due to the limited added value of including PCT, it was not studied further.

The clinical prediction rule based on the GP’s suspected diagnosis on initial consultation reached an AUC of 0.63 (95% CI: [0.61, 0.68]). As the subgroup of randomised patients that was predicted to suffer from pneumonia based on the GP’s clinical judgment consists of only seven patients, this subgroup was not studied further. Addition of CRP to the GP’s suspicion increased the AUC to 0.77 (95% CI: [0.72, 0.81]). Addition of PCT to the GP’s suspicion increased the AUC to 0.69 (95% CI: [0.63, 0.73]).

### 2.3. Evaluation of Treatment Effect

In total, 2056 patients that fulfilled all inclusion criteria were randomised: 1035 patients received amoxicillin, 1021 patients received placebo. Illness deterioration (yes/no) was registered for 2022 patients (98.3%) of whom 354 (17.5%) experienced illness deterioration. The vast majority of those with illness deterioration represented reconsultation rather than hospital admission. Symptom duration and severity of symptoms were reported for 1802 (87.6%) and 1791 (87.1%) patients, respectively. Sample size information for subgroup analyses is presented in Figure 2.

#### 2.3.1. Symptom Duration

Neither patients predicted to have a combined infection nor patients predicted to have pneumonia were significantly more likely to benefit from amoxicillin regarding the duration of symptoms (in days) rated moderately bad or worse (Table 2).

#### 2.3.2. Symptom Severity

Neither patients predicted to have a combined infection nor patients predicted to have pneumonia were significantly more likely to benefit from amoxicillin regarding symptom severity (Table 3).

#### 2.3.3. Illness deterioration

Neither patients predicted to have a combined infection nor patients predicted to have pneumonia were significantly more likely to benefit from amoxicillin regarding illness deterioration (Table 4).

## 3. Discussion

Previous analyses of the GRACE trial (amoxicillin versus placebo in adult acute cough patients in primary care) found that amoxicillin provided little benefit overall and was even associated with slight harm [3]. Secondary subgroup analyses found no clear evidence of clinically meaningful benefit from amoxicillin in high-risk patient groups (e.g., significant comorbidities) [4]. However, additional subgroup analyses detected a significant reduction in symptom duration and symptom severity (at days 2–4, reported by the patient) in patients with pneumonia (based on chest radiograph) [5] and a significant reduction in illness deterioration in patients with a combined viral–bacterial infection (based on modern microbiological methodology) [6] when treated with amoxicillin.

Since the results of chest radiography and microbiological sampling and laboratory testing are not readily available in primary care, clinical prediction rules which predict pneumonia or a combined infection based on signs and symptoms that are available during the initial consultation could be useful to guide antibiotic prescribing in clinical practice.

A clinical prediction rule for pneumonia based on readily available signs and symptoms has been developed by Van Vugt et al. The inclusion of CRP increased the prediction rule’s performance, advocating for CRP assessments during the initial consultation [7]. In this manuscript, a prediction rule for a combined infection using signs and symptoms that can be obtained during the initial consultation was developed. Its performance also increased upon the inclusion of CRP, although it remained suboptimal, hence misclassifying a large portion of the acute cough patients (Figure 2). However, even though previous research has shown that clinical prediction rules are among the more effective methods to reduce inappropriate prescribing of antibiotics for acute respiratory tract infections [10], and the existing prediction rule for pneumonia and the newly developed prediction rule for a combined infection have an excellent and adequate performance, respectively, we found no benefit of amoxicillin treatment in patients that were predicted to suffer from pneumonia or patients predicted to have a combined infection. These findings highlight the need for additional research into quick ways to adequately assess the presence of pneumonia or a combined viral–bacterial infection at the point of care, as these appear to be the most useful indicators for benefit of treatment with amoxicillin in adult uncomplicated acute cough patients.

### Strengths and Limitations

This is the first study to quantify the diagnostic value of signs and symptoms and the additional diagnostic value of inflammatory markers for combined viral–bacterial infections, the first to assess the additional diagnostic value of inflammatory markers to GPs’ suspicions of pneumonia, and the first to assess the effect of amoxicillin in adults presenting to primary care with acute cough predicted to have a pneumonia or a combined infection. The sample size was large, chest radiographs were assessed by radiologists who were blinded from the patient’s clinical investigation, and all blood samples and swabs were analysed in the same laboratory with modern methodology. However, the subgroups studied in this secondary analysis were not defined in advance, and although using a large trial dataset, the study was possibly underpowered to detect interactions between subgroup and antibiotic use [11]. In addition, the prediction rules’ performance was suboptimal, especially for combined infection. Therefore, the findings should be interpreted with caution. Both CRP and PCT were analysed in the lab using conventional blood tests rather than in primary care using point-of-care tests, which calls for a comparison between blood tests used by lab staff and point-of-care tests used by primary care clinicians.

## 4. Materials and Methods

### 4.1. Data

An observational study on the aetiology, diagnosis and prognosis, and a nested RCT on the effect of amoxicillin in adult acute cough patients were conducted between November 2007 and April 2010 within the GRACE Network of Excellence [3,7,9,12]. Patients originated from 16 primary care networks within 12 European countries (Belgium, England, France, Germany, Italy, the Netherlands, Poland, Spain, Slovakia, Slovenia, Sweden and Wales). Eligible patients were individuals aged ≥18 years who consulted their GP with an acute cough (first consultation for this symptom and duration of cough before the consultation maximally 28 days). Exclusion criteria were pregnancy, treatment with antibiotics in the previous month and immunodeficiency.

During the consultation, GPs recorded the patients’ clinical signs (general impression, lung auscultation findings, heart rate, respiratory rate, blood pressure and temperature), baseline symptoms (phlegm, shortness of breath, wheeze, runny nose, fever, chest pain, muscle ache, headache, disturbed sleep, feeling unwell, interference with normal activities or work, confusion or disorientation and diarrhoea), comorbidities (pulmonary comorbidities: chronic obstructive pulmonary disease, asthma, other lung disease (e.g., fibrosis); cardiac comorbidities: heart failure, ischemic heart disease, other heart disease (e.g., cardiomyopathy); and diabetes) and suspected diagnosis on a case report form.

Within 24 h of the consultation, serum and blood, sputum, if available, and two nasopharyngeal swabs were taken which were sent to the University Hospital in Antwerp. Bacteria and viruses were detected using modern microbiological methodology [9]. Bacterial pathogens that were tested for include *Streptococcus pneumoniae*, *Haemophilus influenzae*, *Mycoplasma pneumoniae*, *Chlamydophila pneumoniae*, *Bordetella pertussis* and *Legionella pneumophila (pneumonia).* Viral pathogens that were tested for include rhinoviruses, influenza virus A and B, coronaviruses, respiratory syncytial virus, human metapneumovirus, parainfluenza virus 1–4, adenovirus, polyomavirus and bocavirus. The presence of both a viral and a potential bacterial pathogen was referred to as a combined infection. CRP and PCT were measured with conventional methodology [13].

Within three (maximum seven) days of the consultation, patients were subjected to a chest radiograph which was assessed by radiologists blinded to the patient’s clinical investigation. During the course of their illness (or up to 28 days), all patients filled in a diary scoring their symptoms on a seven point Likert scale (0 = normal/not affected, 1 = very little problem, 2 = slight problem, 3 = moderately bad, 4 = bad, 5 = very bad, 6 = as bad as it could be).

### 4.2. Prediction Rules for Combined Infection

Missing covariate information was imputed using multiple imputation by chained equations (five imputations) [14]. A conditional random forest approach, which splits subgroups in order to maximize the differential effect in terms of the response variable, was used to identify the most important variables for each imputed dataset [15]. Important covariates were selected based on the mean decrease in accuracy. Selected covariates were included in a logistic regression model (pooled over the five imputations). From this full model, non-significant covariates were removed in a backwards fashion (α = 0.05). The prediction rule was based on the final model and its pooled parameter estimates, with the optimal cut-off value determined using the Youden index [16]. Additional prediction rules were constructed by including either CRP or PCT in the final model.

### 4.3. Prediction Rules for Pneumonia

The prediction rule for pneumonia was based on the model by Van Vugt et al. [7] and its pooled parameter estimates, with the optimal cut-off value determined using the Youden index. In addition, a prediction rule based on a model consisting of the GP’s suspected diagnosis on initial consultation was constructed. Additional prediction rules were constructed by including either CRP or PCT in these models.

### 4.4. Prediction Rule Evaluation

The clinical prediction rules’ performance, and its improvement obtained by including biomarker information (CRP and PCT), was evaluated using AUC. Empirical bootstrapping (200 resamples) was used to obtain 95% CIs.

### 4.5. Evaluation of Treatment Effect

All patients in the observational study that were not allergic to penicillin, not suspected of pneumonia and agreed to randomisation were allocated to receive either an antibiotic (amoxicillin 1 g) or a placebo three times a day for seven consecutive days. The effectiveness of amoxicillin treatment in patients predicted to have either pneumonia or a combined infection was assessed using symptom duration, symptom severity and illness deterioration. Symptom duration was defined as the duration of symptoms rated “moderately bad or worse” (one symptom scoring ≥ 3) by patients. Symptom severity was defined as the mean diary score for all symptoms on days 2–4. Illness deterioration was defined as reconsultation with new or worsening complaints or illness necessitating hospital admission within four weeks of the initial consultation.

Analysis used regression models controlling for severity of symptoms at baseline: Cox regression for duration of symptoms allowing for censoring; simple linear regression for symptom severity; and logistic regression for illness deterioration [17,18,19]. Interaction terms were used to estimate the difference in effectiveness of amoxicillin in different subgroups. The subgroups of interest were patients predicted to have a pneumonia (by the GP or by a prediction rule) and patients predicted to have a combined infection (by a prediction rule).

## 5. Conclusions

While adults presenting to primary care with acute cough that are diagnosed with pneumonia (based on chest radiograph) or a combined viral–bacterial infection (based on modern microbiological methodology) benefit from treatment with amoxicillin, we did not find any benefit on symptom duration, symptom severity or illness deterioration in patients where these diagnoses are based on clinical prediction rules, regardless of including biomarker information (CRP or PCT). The studied prediction rules may have a place in helping primary care clinicians to reduce antibiotic prescribing, but this study provides no evidence that using the prediction rules will adequately identify adult acute cough patients that will benefit from amoxicillin treatment in primary care.

## Figures and Tables

**Table 1:** Overview of patient characteristics and their association with the odds of a combined infection in adults presenting to primary care with acute cough.

Patient Characteristics	Number (%) of Patients ^a^ (*n* = 3104)	Number (%) with Missing Information	Patients with Combined Infection (*n* = 304)	
			Number (%) ^a^	OR (95% CI)
**General characteristics**				
Age (years): mean ± SD	49.8 ± 16.8	0 (0)	48.5 ± 16.6	0.99 [0.99–1.00]
Male	1244 (40.1)	0 (0)	122 (40.1)	1.00 [0.79–1.27]
Current smoker	871 (28.1)	3 (0.1)	97 (31.9)	1.22 [0.95–1.58]
No. of days coughing before consultation: mean ± SD	8.7 ± 7.4	46 (1.5)	7.5 ± 5.9	0.97 [0.95–0.99]
No. of days illness before consultation: mean ± SD	9.7 ± 10.2	31 (1.0)	8.1 ± 6.1	0.97 [0.95–0.99]
**Clinical signs**				
Abnormal consciousness	44 (1.4)	3 (0.1)	5 (1.6)	1.18 [0.41–2.76]
General toxicity	800 (25.8)	8 (0.3)	92 (30.3)	1.28 [0.98–1.65]
Lung auscultation:				
Diminished vesicular breathing	393 (12.7)	20 (0.6)	48 (15.9)	1.34 [0.96–1.85]
Wheeze	539 (17.5)	21 (0.7)	65 (21.7)	1.35 [1.00–1.79]
Crackles	289 (9.4)	18 (0.6)	30 (10.0)	1.08 [0.71–1.58]
Rhonchi	521 (16.9)	21 (0.7)	65 (21.6)	1.40 [1.04–1.87]
Tachycardia (>100 beats/min)	85 (2.8)	44 (1.4)	7 (2.3)	0.82 [0.34–1.68]
Tachypnoea (>24 breaths/min)	61 (2.0)	78 (2.5)	8 (2.7)	1.39 [0.61–2.79]
Prolonged expiration	309 (10.1)	45 (1.4)	44 (14.8)	1.64 [1.15–2.29]
Low blood pressure (<90/60 mmHg)	10 (0.3)	67 (2.2)	0 (0)	0.44 [0.00–3.44] ^d^
Fever (oral temperature >37.8 °C)	137 (4.5)	31 (1.0)	18 (6.1)	1.45 [0.84–2.35]
**Baseline symptoms** (as reported by the patient during consultation)				
Phlegm	2472 (79.7)	4 (0.1)	252 (83.2)	1.28 [0.95–1.78]
Shortness of breath	1754 (56.6)	4 (0.1)	180 (59.4)	1.14 [0.89–1.45]
Wheeze	1324 (42.7)	5 (0.2)	139 (45.9)	1.15 [0.91–1.46]
Runny nose	2212 (71.4)	4 (0.1)	235 (77.6)	1.43 [1.09–1.91]
Fever	1085 (35.0)	5 (0.2)	133 (43.9)	1.52 [1.19–1.93]
Chest pain	1433 (46.3)	6 (0.2)	151 (49.8)	1.17 [0.92–1.49]
Muscle ache	1573 (50.7)	4 (0.1)	170 (56.1)	1.27 [1.00–1.61]
Headache	1742 (56.2)	3 (0.1)	191 (62.8)	1.36 [1.07–1.74]
Disturbed sleep	1955 (63.1)	5 (0.2)	207 (68.1)	1.28 [1.00–1.65]
Myalgia	2349 (75.8)	4 (0.1)	244 (80.5)	1.36 [1.02–1.84]
Interference with daily activities	1955 (63.0)	3 (0.1)	212 (69.7)	1.39 [1.08–1.81]
Confusion/disorientation	137 (4.4)	4 (0.1)	17 (5.6)	1.32 [0.76–2.17]
Diarrhoea	222 (7.2)	4 (0.1)	24 (7.9)	1.12 [0.71–1.71]
**Comorbidities**				
Pulmonary comorbidity ^b^	528 (17.0)	2 (0.1)	49 (16.1)	0.93 [0.67–1.27]
Cardiac comorbidity ^c^	288 (9.3)	3 (0.1)	27 (8.9)	0.95 [0.61–1.41]
Diabetes	200 (6.5)	4 (0.1)	14 (4.6)	0.68 [0.37–1.14]
Previous hospitalisation for respiratory illness	129 (4.2)	2 (0.1)	14 (4.6)	1.13 [0.61–1.92]
Antibiotic treatment in previous six months	460 (14.8)	1 (0.0)	34 (11.2)	0.70 [0.48–1.00]
Allergic disease	562 (18.1)	5 (0.2)	59 (19.4)	1.10 [0.81–1.47]
**Other regular medication**				
Inhaled bronchodilators	348 (11.2)	2 (0.1)	33 (10.9)	0.96 [0.65–1.38]
Inhaled steroids	270 (8.7)	2 (0.1)	26 (8.6)	0.98 [0.63–1.47]
Oral steroids	43 (1.4)	1 (0.0)	2 (0.7)	0.45 [0.07–1.46]
Oral agents for diabetes	153 (4.9)	1 (0.0)	9 (3.0)	0.56 [0.26–1.05]
Insulin	45 (1.5)	1 (0.0)	4 (1.3)	0.90 [0.27–2.24]
Antihypertensives/diuretics	734 (23.7)	2 (0.1)	66 (21.7)	0.88 [0.66–1.17]
Nonsteroidal anti-inflammatories	251 (8.1)	1 (0.0)	26 (8.6)	1.07 [0.68–1.61]
Benzodiazepines/antidepressants	307 (9.9)	1 (0.0)	25 (8.2)	0.80 [0.51–1.20]
Influenza vaccination received this autumn/winter	732 (23.6)	2 (0.1)	62 (20.4)	0.81 [0.60–1.08]
**Blood test results**				
C-reactive protein: mean ± SD	1.8 ± 3.5	174 (5.6)	3.1 ± 4.8	1.08 [1.06–1.11]
Procalcitonin: mean ± SD	0.06 ± 0.22	164 (5.3)	0.10 ± 0.66	1.99 [1.10–5.17]

SD = standard deviation; CI = confidence interval; OR = odds ratio; ^a^ Except where indicated otherwise. ^b^ Pulmonary comorbidities = chronic obstructive pulmonary disease + asthma + other lung disease (e.g., fibrosis); ^c^ Cardiac comorbidities = heart failure + ischemic heart disease + other heart disease (e.g., cardiomyopathy); ^d^ Odds ratios obtained through Firth corrected logistic regression (because of quasi-complete separation).

**Table 2:** Median symptom duration ^a^ (interquartile range) in patients presenting to primary care with acute cough treated with amoxicillin versus placebo.

	Amoxicillin	Placebo	Interaction Term ^b^ (95% CI)	*p*-Value	Hazard Ratio for Subgroup ^b^ (95% CI)	*p*-Value
WHOLE COHORT (*N* = 1802)	6 (3–11)	7 (3–13)			1.06 [0.96, 1.17]	0.278
COMBINED INFECTION:						
PREDICTED ^c^ (*N* = 1307)	6 (3–11)	7 (4–12)	1.01 [0.80, 1.27]	0.945	1.07 [0.95, 1.20]	0.287
PREDICTED_CRP ^c^ (*N* = 233)	8 (4–16)	8 (5–14)	0.87 [0.64, 1.17]	0.351	0.90 [0.68, 1.20]	0.474
PNEUMONIA:						
PREDICTED ^d^ (*N* = 225)	7 (4–15)	7 (5–11)	0.83 [0.61, 1.11]	0.207	0.88 [0.67, 1.16]	0.370
PREDICTED_CRP ^d^ (*N* = 216)	7 (4–15)	7 (5–11)	0.78 [0.57, 1.07]	0.119	0.80 [0.59, 1.07]	0.126
PREDICTED_GP_CRP ^e^ (*N* = 210)	8 (4–14)	8 (5–13)	1.03 [0.75, 1.41]	0.851	1.04 [0.77, 1.39]	0.818
PREDICTED_GP_PCT ^e^ (*N* = 138)	6 (3–14)	7 (3–13)	0.80 [0.55, 1.17]	0.250	0.81 [0.56, 1.17]	0.260

CI, confidence interval; CRP, C-reactive protein; GP, general practitioner’s suspected diagnosis; PCT, procalcitonin; ^a^ calculated as number of days with symptoms rated moderately bad or worse by the patient after the initial consultation; ^b^ estimates are controlled for baseline symptom severity; ^c^ patients predicted to have a combined infection based on the prediction rule constructed in this manuscript without (predicted) and with (predicted_CRP) inclusion of CRP; ^d^ patients predicted to have a pneumonia based on the prediction rule constructed by Van Vugt et al. without (predicted) and with (predicted_CRP) inclusion of CRP; ^e^ patients predicted to have a pneumonia based on the GP’s suspected diagnosis with inclusion of CRP (predicted_GP_CRP) or PCT (predicted_GP_PCT).

**Table 3:** Mean symptom severity ^a^ (standard deviation) in patients presenting to primary care with acute cough treated with amoxicillin versus placebo.

	Amoxicillin	Placebo	Interaction Term ^b^ (95% CI)	*p*-Value	Difference for Subgroup ^b^ (95% CI)	*p*-Value
WHOLE COHORT (*N* = 1791)	1.59 (0.96)	1.70 (1.01)			−0.07 [−0.15, 0.00]	0.065
COMBINED INFECTION:						
PREDICTED ^c^ (*N* = 1298)	1.73 (0.97)	1.83 (1.01)	0.00 [−0.17, 0.18]	0.968	−0.06 [−0.16, 0.03]	0.187
PREDICTED_CRP ^c^ (*N* = 233)	2.15 (1.04)	2.21 (1.03)	0.10 [−0.13, 0.33]	0.384	0.02 [−0.24, 0.27]	0.907
PNEUMONIA:						
PREDICTED ^d^ (*N* = 225)	1.93 (0.99)	1.95 (1.02)	0.12 [−0.12, 0.35]	0.321	0.03 [−0.19, 0.25]	0.783
PREDICTED_CRP ^d^ (*N* = 216)	2.06 (1.02)	2.09 (1.12)	0.09 [−0.15, 0.33]	0.451	0.02 [−0.23, 0.28]	0.852
PREDICTED_GP_CRP ^e^ (*N* = 209)	1.97 (0.97)	2.17 (1.07)	-0.07 [−0.32, 0.17]	0.554	−0.14 [−0.40, 0.12]	0.298
PREDICTED_GP_PCT ^e^ (*N* = 138)	1.72 (0.88)	1.85 (1.12)	-0.06 [−0.36, 0.23]	0.684	−0.12 [−0.41, 0.17]	0.417

CI, confidence interval; CRP, C-reactive protein; GP, general practitioner’s suspected diagnosis; PCT, procalcitonin; ^a^ calculated as the mean diary score for all symptoms on days 2-4 (rated by the patient); ^b^ estimates are controlled for baseline symptom severity; ^c^ patients predicted to have a combined infection based on the prediction rule constructed in this manuscript without (predicted) and with (predicted_CRP) inclusion of CRP; ^d^ patients predicted to have a pneumonia based on the prediction rule constructed by Van Vugt et al. without (predicted) and with (predicted_CRP) inclusion of CRP; ^e^ patients predicted to have a pneumonia based on the GP’s suspected diagnosis with inclusion of CRP (predicted_GP_CRP) or PCT (predicted_GP_PCT).

**Table 4:** Illness deterioration ^a^ in patients consulting in primary care with acute cough treated with amoxicillin versus placebo.

	Amoxicillin	Placebo	Interaction Term ^b^ (95% CI)	*p*-Value	Odds Ratio for Subgroup ^b^ (95% CI)	*p*-Value
WHOLE COHORT (*N* = 2022)	162/1018	192/1004			0.81 [0.64, 1.02]	0.073
COMBINED INFECTION:						
PREDICTED ^c^ (*N* = 1471)	117/743	134/728	1.08 [0.64, 1.82]	0.785	0.84 [0.64, 1.10]	0.207
PREDICTED_CRP ^c^ (*N* = 263)	27/131	28/132	1.26 [0.65, 2.41]	0.492	0.98 [0.54, 1.79]	0.952
PNEUMONIA:						
PREDICTED ^d^ (*N* = 245)	20/117	26/128	1.02 [0.50, 2.03]	0.963	0.82 [0.42, 1.56]	0.542
PREDICTED_CRP ^d^ (*N* = 243)	21/113	27/130	1.11 [0.55, 2.21]	0.762	0.87 [0.46, 1.64]	0.665
PREDICTED_GP_CRP ^e^ (*N* = 237)	20/115	27/122	0.93 [0.46, 1.86]	0.839	0.75 [0.39, 1.41]	0.381
PREDICTED_GP_PCT ^e^ (*N* = 159)	16/86	19/73	0.81 [0.36, 1.79]	0.602	0.65 [0.30, 1.38]	0.262

CI, confidence interval; CRP, C-reactive protein; GP, general practitioner’s suspected diagnosis; PCT, procalcitonin; ^a^ defined as a return to the physician with worsening symptoms, new symptoms, new signs, or illness requiring admission to hospital within 4 weeks of the initial consultation (determined through a notes review); ^b^ estimates are controlled for baseline symptom severity; ^c^ patients predicted to have a combined infection based on the prediction rule constructed in this manuscript without (predicted) and with (predicted_CRP) inclusion of CRP; ^d^ patients predicted to have a pneumonia based on the prediction rule constructed by Van Vugt et al. without (predicted) and with (predicted_CRP) inclusion of CRP; ^e^ patients predicted to have a pneumonia based on the GP’s suspected diagnosis with inclusion of CRP (predicted_GP_CRP) or PCT (predicted_GP_PCT).

## Data Availability

All GRACE data are available upon motivated request from the project coordinator, Herman Goossens, according the project’s standard operating procedures.
